# Case report: Craniofrontonasal syndrome caused by a novel variant in the *EFNB1* gene in a Colombian woman

**DOI:** 10.3389/fgene.2022.1092301

**Published:** 2023-01-04

**Authors:** Harry Pachajoa, Diana Marcela Vasquez-Forero, Sebastian Giraldo-Ocampo

**Affiliations:** ^1^ Genetics Division, Fundación Valle del Lili, Cali, Colombia; ^2^ Centro de Investigaciones en Anomalías Congénitas y Enfermedades Raras (CIACER), Universidad Icesi, Cali, Colombia; ^3^ Departamento de Microbiología, Universidad del Valle, Cali, Colombia

**Keywords:** frontonasal dysplasia, craniosynostosis, hypertelorism, EFNB1 gene, case report

## Abstract

Craniofrontonasal Syndrome is a very rare dominant X-linked genetic disorder characterized by symptoms such as hypertelorism, craniosynostosis, eye alterations, bifid nose tip, and longitudinal ridging and splitting of nails. Heterozygous females are usually the patients severely affected. To date, clinical or genetic data have not been published for these patients in Colombia. Here we report a female proband with coronal craniosynostosis, hypertelorism, strabismus, rotational nystagmus, high-arched palate, dental crowding, scoliosis, severe pectus excavatum, unilateral breast hypoplasia, and brachydactyly; diagnosed with Craniofrontonasal Syndrome with the novel heterozygous variant c.374A>C (p.Glu125Ala) in the *EFNB1* gene. So far, she has been treated with physical therapy and surgical correction of the bifid nose and an umbilical hernia. To the best of our knowledge, this is the first report of a patient with this rare genetic disorder in Colombia, expanding its mutational spectrum and highlighting the importance of genetic evaluation of patients with craniosynostosis and facial dysmorphism.

## Introduction

Craniofrontonasal Syndrome (CFNS; OMIM: 304110), also known as craniofrontonasal dysplasia, is a rare, X-linked, developmental disorder characterized by its unusual and paradoxical sex reversal in phenotypic severity: heterozygous females are more affected than hemizygous males (Twigg et al., 2006). Typical clinical manifestations in females include coronal synostosis, leading to the characteristic facial asymmetry, wide-set eyes (hypertelorism), bifid nasal tip, longitudinal ridging and splitting of nails, and wiry curly hair (Van Den Elzen et al., 2014). Other reported manifestations are clinodactyly, cutaneous syndactyly, unilateral breast hypoplasia, bilateral cleft lip and palate, depressed nasal bridge, short and wide upper face, skeletal abnormalities, visual complications, umbilical and diaphragmatic hernia, and corpus callosum agenesis or dysgenesis, among others (Van Den Elzen et al., 2014; Inoue et al., 2018; Acosta-Fernández et al., 2020; Gürsoy et al., 2021). Male carriers commonly present only a few mild signs, such as hypertelorism, or no signs at all (Van Den Elzen et al., 2014).

CFNS is caused by loss-of-function mutations in the *ephrin-B1* gene (*EFNB1,* OMIM: 300035), located in the Xq13.1 region (Twigg et al., 2004; Wieland et al., 2004). The protein coded by this gene, ephrin-B1, binds to the Eph-related receptor tyrosine kinases, where bidirectional signaling of these molecules has been proven to participate in cell adhesion, migration, and pattern formation during embryonic development, particularly in neural crest cell migration and demarcation of the future coronal suture, axon guidance of the corpus callosum and normal craniofacial development (Davy et al., 2004; Bush and Soriano, 2010). The prevalence of CFNS is currently unknown, however, it is considered a very rare congenital disorder, with the largest case series reported, with confirmed *EFNB1* mutations, having 33 patients (Wieland et al., 2005). More case reports and case series are needed to expand the clinical phenotype and mutational spectrum of CFNS, especially in geographical regions where this genetic disorder has not been previously reported. Here we report a Colombian female patient with proven CFNS caused by a novel heterozygous missense variant.

## Case description

A 54-year-old female patient was born to non-related healthy parents. The perinatal clinical history was not available. The patient has a first-degree maternal male cousin with intellectual disability and cleft lip palate. Other family history is unremarkable.

Since childhood, the patient has had several pathologies: an umbilical hernia (surgically corrected), several periodontal infections, proneness to tooth loss, scoliosis (treated only with physical therapy), anemia, dysmenorrhea, learning difficulty (as reported by herself), and left eye refractive error. At 50 years of age, the proband underwent a hysterectomy for endometriosis. Before this procedure, she was never pregnant by her own decision and has no children. At 51 years old, visual acuity evaluation showed that the left (before and after the cover test:20/200, 20/80, respectively) and right eye (before and after the cover test: 20/25, 20/20, respectively) had refractive defects, with the latter more affected. Ophthalmology evaluation revealed hypertelorism, rotational nystagmus, and strabismus (endotrophic left eye).

**FIGURE 1:**
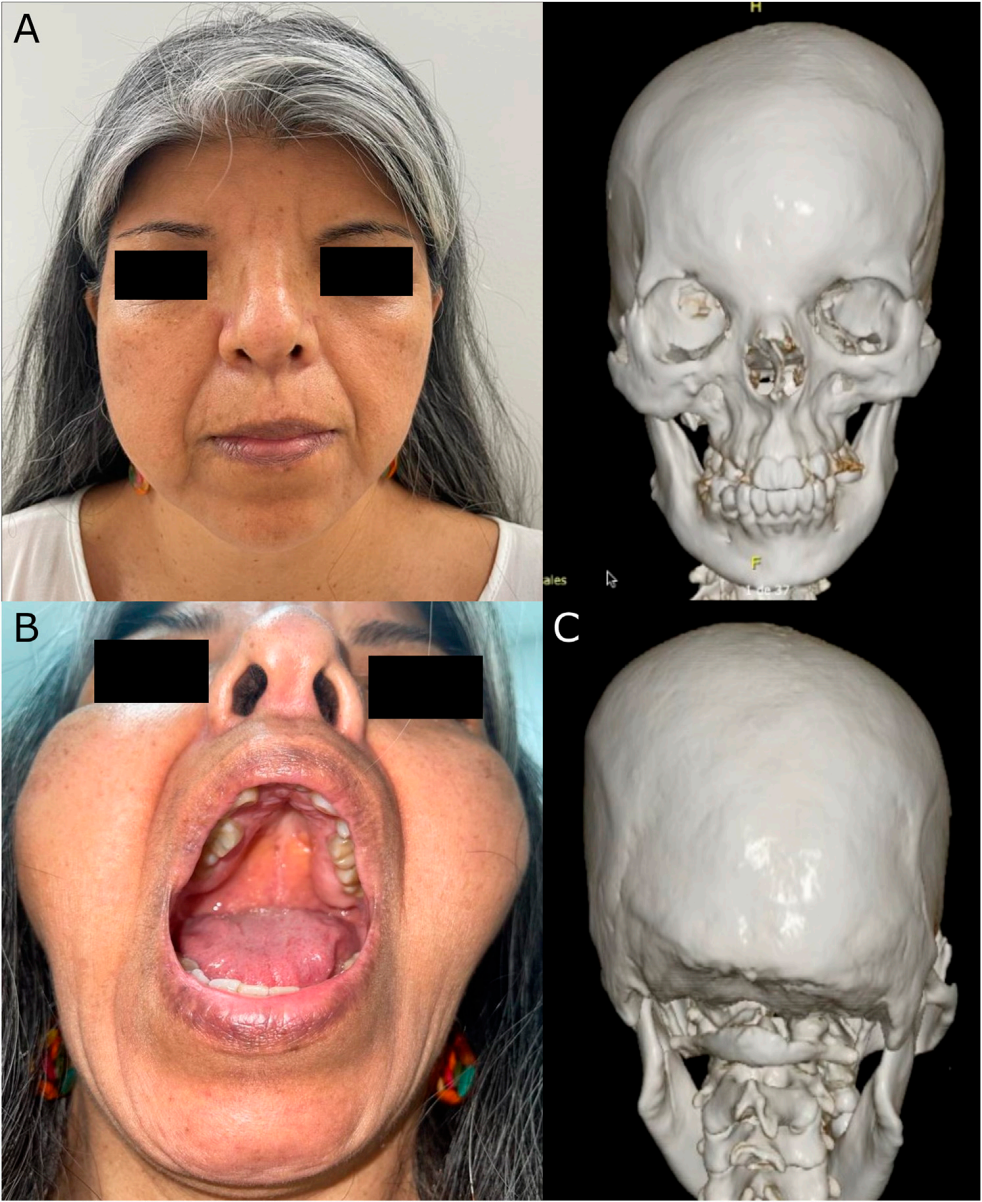
Cranial manifestations in the 54-year-old CFNS female patient. The figure shows **(A)** Facial dysmorphism (left) and frontal view of the 3D-CT showing facial asymmetry, dental crowding and the sinusoidal nasal septum with a large right bone spur (right); **(B)** high-arched palate, and **(C)** posterior view of the 3D-CT showing flat occiput and left parietal bone prominence.

**FIGURE 2:**
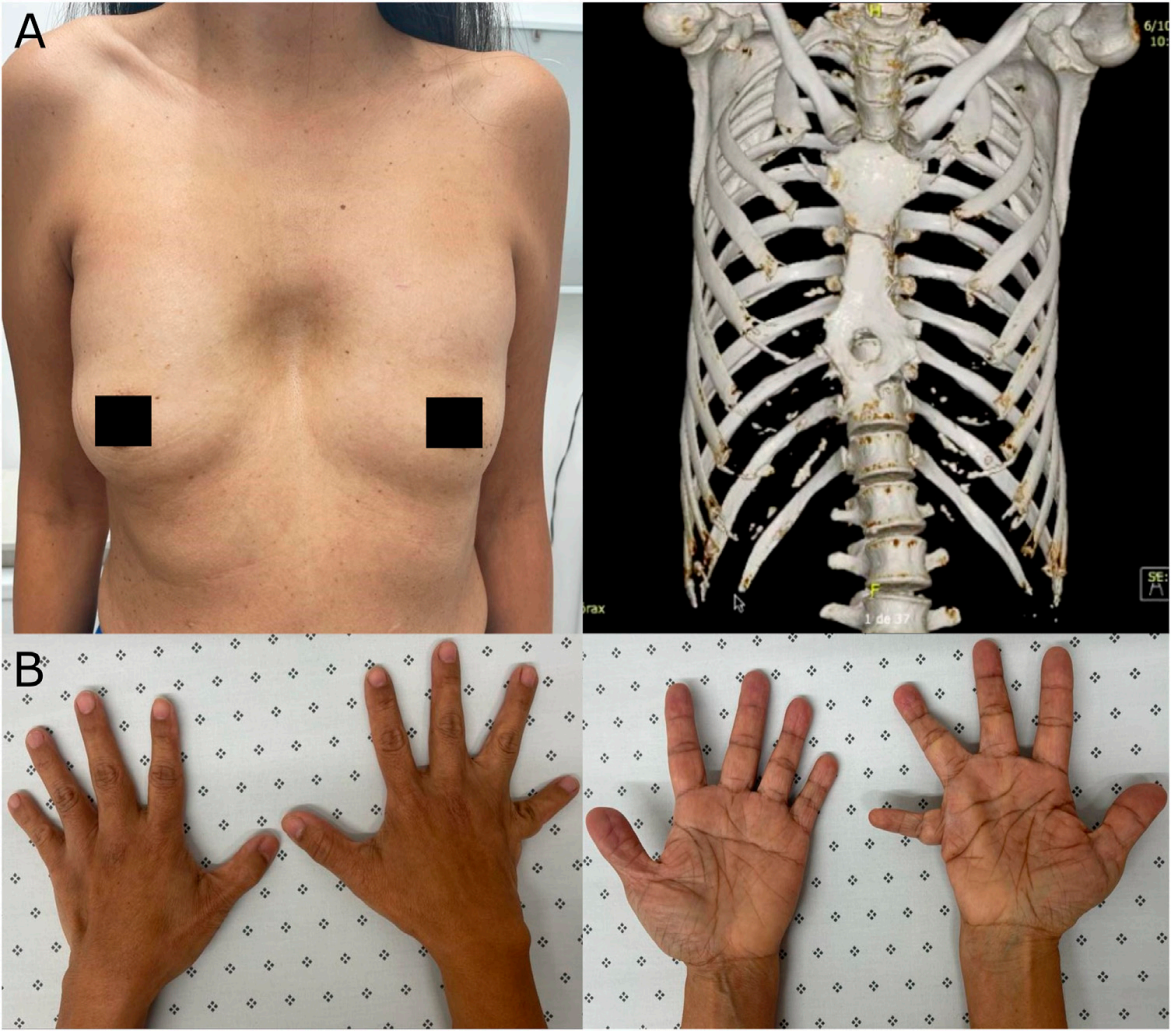
Extracranial manifestations in the patient. **(A)** Unilateral breast hypoplasia (left) and thorax 3D-CT revealing pectus excavatum (right). **(B)** Brachydactyly, a low-set fifth finger of the right hand with hypoplastic metacarpal, and a single left transverse palmar crease.

At 53 years old, the patient attended a physical examination by clinical genetics for the first time, revealing brachycephaly, flat occiput, midface hypoplasia, wide nasal bridge, bifid nasal tip (surgically corrected), hypertelorism, downslanting palpebral fissures, high-arched palate, dental crowding (Figure 1), severe pectus excavatum, unilateral breast hypoplasia, brachydactyly, a low-set fifth finger of the right hand with hypoplastic metacarpal, and a single left transverse palmar crease (Figure 2). The patient did not have longitudinal ridging and splitting of nails. A three-dimensional computed tomography scan (3D-CT) of the skull showed coronal craniosynostosis, sinusoidal nasal septum with a large right bone spur, mucosal thickening of the maxillary and ethmoidal sinuses, lack of pneumatization and poor development of frontal sinuses, and left parietal bone prominence (Figure 1). Thorax CT confirmed the diagnosis of pectus excavatum (Figure 2). Cranial tomography showed no nervous system alterations, an echocardiogram test was within normal range and renal echography did not show renal involvement. Uterine defects were not possible to study given the hysterectomy procedure performed on the patient, and other alterations such as bifid uvula were not noticed during the physical examination. Karyotype results showed a karyotype 46, XX and no chromosophaties. Despite her learning difficulty (as reported by herself), she went to college and has a bachelor’s degree in education. Furthermore, during the interview normal intelligence was noticed, however, a formal IQ evaluation was not done. Her height was 162 cm (42nd percentile) and her weight was 54 kg (30th percentile). The patient reported that about 5 years before the evaluation by clinical genetics, chronic pain in the left hemibody started and is treated to date with physical therapy. Given the clinical manifestations, a diagnosis of craniofrontonasal syndrome (CFNS) was suspected, and therefore, whole exome sequencing with subsequent validation by sanger sequencing was performed. The test revealed the heterozygous variant c.374A>C (p.Glu125Ala) in the *EFNB1* gene (OMIM: 300035) classified as probably pathogenic according to the College of Medical Genetics and Genomics ACMG criteria (Richards et al., 2015), allowing the diagnosis of CNFS in the proband. This variant has been reported neither in the literature nor in the Clinvar, gnomAD, 1,000 genomes, or HGMD databases. This variant is predicted to be deleterious by SIFT (0), and probably damaging by Polyphen2 (0.99). Its DANN and CADD scores are 0.99 and 27.1, respectively.

## Discussion

The neural crest cells participate in the frontonasal process that gives raise to the midline tissue such as frontal bones, the nasal bridge, and the nasal tip during embryonic development (Farlie et al., 2016). These frontonasal crest cells migrate to the prospective frontonasal region of the developing face. Alterations in genes involved in the growth or patterning of these cells can cause abnormal midface development, which clinically is known as frontonasal dysplasia (Farlie et al., 2016).

A distinctive subgroup of frontonasal dysplasia is CFNS, that aside from frontonasal alterations, also shows cranial malformations, particularly coronal synostosis or craniosynostosis (Van Den Elzen et al., 2014). Before 2004, when the molecular etiology of CFNS was reported, several cases of this disorder had already been reported. However, when genetic evaluation of some of those patients was performed, about 20% did not have mutations in the *EFNB1* gene, responsible for CFNS, leading to the conclusion that some of the patients were misdiagnosed and their clinical data could be misleading (Van Den Elzen et al., 2014). The patient described in this report is a proven case of CFNS, given that a likely pathogenic variant was found in the *EFNB1* gene and she presented several typical CFNS symptoms such as coronal synostosis, facial asymmetry, hypertelorism, bifid nasal tip (surgically corrected), unilateral breast hypoplasia, malformation of the palate, visual complications, and an umbilical hernia (Figure 1), but not longitudinal ridging and splitting of nails, a typical CFNS manifestation. Thus, this report adds and supports data about the clinical manifestations of proven CFNS patients and, to the best of our knowledge, this is the first report about this rare genetic disorder in Colombia. Furthermore, an apparent ulnar defect is present on the right hand of the patient (Figure 2B), which together with the unilateral breast hypoplasia (Figure 2A) could suggest an interesting ulnar-mammary manifestation in CFNS patients; but no X-ray was performed on the hands of the patient, and therefore no conclusion can be drawn. Nevertheless, CFNS patients have been reported to present with ulnar alterations (Twigg et al., 2006) or unilateral breast hypoplasia (Van Den Elzen et al., 2014). Thus, the manifestation of both symptoms in the same patient is possible.

About 123 variants in the *EFNB1* gene, almost all missense, non-sense, and frameshift, have been reported in patients with CFNS (Bukowska-Olech et al., 2021), with the majority of them located at exons 2 and 3 (of the five exons this gene has) that code for the extracellular ephrin domain and thus, affecting the interaction between the ephrin-B1 protein and the Eph receptors (Darling and Lamb, 2019; Gürsoy et al., 2021). Accordingly, the variant c.374A>C (p.Glu125Ala) reported in our patient is located at exon 2, changing a polar (glutamic acid) for a non-polar aminoacid (alanine) and probably affecting the receptor-binding domain of the protein, leading to the clinical manifestations described in this proband.

Changes in residue 125 of ephrin-B1 have been already reported in a young patient with CFNS in which the glutamic acid changed to lysine (c.373G>A, p.Glu125Lys). This patient showed skeletal deformity of the right orbit, hypotropia of the left eye, hypertelorism, synostosis of the right coronal suture, and a bony cleft of the right nasal bone (Apostolopoulou et al., 2012). Similar symptoms were found in our patient: synostosis, hypertelorism, and strabismus; however, these are typical manifestations and probably not a genotype-phenotype correlation, as this relationship has not been possible to be established for CFNS patients (Van Den Elzen et al., 2014). Furthermore, residue 125 of this protein is conserved between human, mouse, and rat, suggesting that changes in this position are not well tolerated as observed by the phenotype in our patient and the young girl patient mentioned above.

A striking point of this particular case is the age at diagnosis (53 years old) that for CFNS is usually in the first two decades of life (Van Den Elzen et al., 2014). The delay in diagnosis in our patient probably reflects the lack of awareness of genetic disorders that involve facial and cranial alterations. Craniosynostosis, presented in the proband, has a prevalence of 1 per 2,250 births, and some of the cases can be presented as part of a syndrome. Genetic evaluation (exome or genome analysis) of these patients has proven to be beneficial, as 57 genes have already been associated with craniosynostosis and about 37% of patients have a genetic cause (Miller et al., 2017). This case also highlights the importance of genetic evaluation in patients with craniosynostosis with concurrence facial dysmorphia.

In conclusion, we report a proven case of Craniofrontonasal Syndrome (CFNS) with the typical clinical manifestations and the novel heterozygous variant c.374A>C (p.Glu125Ala) in the *EFNB1* gene. Whether other symptoms seen in our patient that are not commonly reported in patients with CFNS such as susceptibility to periodontal infections and chronic pain in left hemibody are CFNS-associated remains unknown. To the best of our knowledge, this is the first case of CFNS reported in Colombia. It supports the reported clinical manifestations of this disorder, and its associated medical problems but also expands its mutational spectrum. This case also highlights the importance of genetic evaluation of patients with craniosynostosis and facial dysmorphism.

## Data Availability

The raw data supporting the conclusions of this article will be made available by the authors, without undue reservation.
